# Expression of Apoptosis Related and Proliferative Proteins in Malignant Lympho-Proliferative Disorders

**Published:** 2017-05-30

**Authors:** Zeeba S Jairajpuri, Rekha Ghai, Sumita Saluja, Sujala Kapur, K.T Bhowmik

**Affiliations:** 1 *Dept* *.* * of Pathology, Hamdard Institute of Medical Sciences and Research, Jamia Hamdard, New Delhi, India*; 2 *National Institute of Pathology, Indian Council Of Medical Research, Safdarjung Hospital Complex, New Delhi India*; 3 *Dept. of Haematology Vardhman Mahavir Medical College and Safdarjung Hospital, New Delhi, India*; 4 *Dept* *.* * of Radiotherapy, Vardhman Mahavir Medical College and Safdarjung Hospital, New Delhi, India*

**Keywords:** Apoptosis, Malignant lymphoproliferative, Bcl2, Bax

## Abstract

**Background & Objective:**

The current study aimed to perform an immunohistochemical analysis of patterns of apoptotic and cell proliferative related protein expression in different histological grades and immune phenotypes of malignant lymphomas and other lymphoproliferative disorders

**Methods:**

This observational study was carried on 60lymph node biopsies of lymphoproliferative disorders. The biopsies were analyzed histologically and immunohistochemically.

**Results:**

A total of 60 lymph node biopsies were included in the study, of which 81.6% were of malignant lympho-proliferative lesions. The majority of the biopsies were B-cell (66%) and were grouped in the intermediate grade. Bax and BCL-2 protein expression was presented by percentage of immune positive neoplastic cells per 10fields and graded on a scale of 1 to4. A Bcl-2, Bax Protein Ratio (BBPR) was determined for each case by dividing the estimated Bcl-2 protein (percentage of Bcl-2 positive cells x Bcl-2 staining intensity) by the estimated Bax protein (percentage of Bax positive cells x Bax immunostaining intensity). The mean BBPR was found to be significantly higher in indolent lymphomas (2.64 ± 1.3) as compared to aggressive lymphomas (0.47 ± 0.9) (P<0.01). The expression of P53 and PCNA in 35 biopsies of Non Hodgkin Lymphomas (NHL) was found to increase from low to high grade tumors.

**Conclusions:**

A significant correlation was found between BBPR and predicted biological behavior of indolent and aggressive lymphomas. This indicates the important role of Bcl-2 and Bax in biological behavior of lymphomas. Furthermore, P53 and PCNA expression were found to increase from low to high-grade tumors suggesting their prognostic value in NHL.

## Introduction

The histological spectrum of benign and malignant lymphoproliferative disorders range from non-specific reactive hyperplasia to atypical lymphoid hyperplasia and lymphomas (Non-Hodgkin’s Lymphoma (NHL) and Hodgkin’s Disease (HD) (1). Molecular, biological and genetic discoveries have improved the primary diagnosis of lymphoproliferative processes permitting better assessment of prognosis within histologic groups (2). Furthermore, integration of clinical features with recent advances in lymphoma biology has contributed significantly to the understanding of tumor specific variables that have a direct impact on clinical behaviors. Integration of clinical features with morphology, cytochemistry and immune phenotype has clinical and prognostic value (3).A key issue regarding lymphoid neoplasm is to discriminate between disease entities and prognostic factors. Both apoptosis and mitosis serve as potential prognostic indicators and are firmly interrelated. The growth of both indolent and aggressive lymphomas depends on the proliferation and death rates of cancer cells. Cell proliferation, as determined by immunocytochemistry, using proliferation antigens Ki-67 and Proliferating Cell Nuclear Antigen (PCNA), is the principal determinant of tumor progression and prognosis. Apoptosis or programmed cell death on the other hand, occurs through a sequence of active mechanisms within the cell that are controlled by positive and negative regulators of the apoptotic pathway (4). A number of genes including c-myc, P53, Bcl-2 and Bax are known to regulate the apoptotic pathway with some preventing and others promoting cell death. The failure of apoptosis leads to the development of many tumors and often renders them resistant to cytotoxic therapies. In hematologic malignancies, this impairment of apoptosis is often caused by overexpression of the pro-survival protein Bcl-2. Abnormally high levels of Bcl-2 help sustain tumors and can be used as a target in an approach to treat various hematologic malignancies (5).

 Expression of Bcl-2 plays an important role in carcinogenesis and was found to be over-expressed in various epithelial tumors. In addition, the expression of apoptosis suppressing Bcl-2 is frequently detected in chronic lymphocytic leukemia, some acute leukemia as well as in NHL, where it appears to be a significant prognostic marker. Bax is a potent promoter of apoptotic pathway and high Bax expression has been documented in lymphoproliferative disorders (6).

The current study performed an immunohistochemical analysis of apoptotic and cell proliferative-related protein expression patterns in different histological grades and immune phenotypes of malignant lymphomas and other lymphoproliferative disorders.

## Materials and Methods

The present study was an observational study conducted on 60 lymph node biopsies of lymphoproliferative disorders, at the National Institute of Pathology, New Delhi. Paraffin-embedded biopsy sections of all age groups with diagnosis of malignant lymphoma (NHL and HD), were included along with extra nodal lymphomas. Four-micron-thick sections, stained with haematoxylinand eosin, were evaluated. Initial diagnoses of benign or malignant lymphoproliferative disease were made and further confirmation along with histological typing were based on immunohistochemical analysis.

A panel of antibodies was used to classify all patients with lymphomas, according to working formulation and REAL/World Health Organization (WHO) classification. Concentration of each primary antibody was standardized and suitable controls were used for each batch of experiments. The antibodies included Leucocyte Common Antigen (LCA), CD 3 and CD 45 RO (for T-cell lymphomas), CD 20 (for B-cell lymphomas), and CD15 and CD 30 (for anaplastic large cell lymphomas and HD). All antibodies were procured from Dakopatts, Copenhagen. Furthermore, expression pattern of proliferative- and apoptotic-related proteins were evaluated. Bcl-2 and Bax were used as primary antibodies for apoptotic-related proteins. Evaluation of staining intensity of tumor cells was done for quantification of immunohistochemistry in the tumor area. The mean was calculated according to the method described by Wheaton et al. (7).The fraction of stained tumor cells was determined. Quantification of immunohistochemistry in the tumor area was done by grading intensity of staining as negative (0), weakly positive (1), moderately positive (2) and strongly positive (3). The fraction of stained tumor cells was categorized to 3 categories: 0 for <25%, 1 for 25% to 75%, and 2 for >75% of stained cells. For each case, the values of the two parameters were added resulting in Bcl-2 and Bax scores ranging from 0 to 5. To assess the propensity of tumors to undergo apoptosis, the ratio of Bcl-2: Bax was calculated. The Bcl-2, Bax Protein Ratio (BBPR) was determined for each case by dividing the estimated Bcl-2 protein (percentage of Bcl-2 positive cells x Bcl-2 staining intensity) by the estimated Bax protein (percentage of Bax positive cells x Bax immunostaining intensity). Percentage of positive cells and intensity of Bcl-2 and Bax protein expression as well as BBPR for each case was recorded. Statistical analysis was performed using Student’sttest to determine significant differences in BBPR values. P values of < 0.05 were considered significant. For proliferative index, PCNA antibody was used and cells showing nuclear positivity were regarded as positive. Approximately 500 tumor cells were counted and PCNA labeling index was calculated as a percentage of positive cells. These results were given as mean ± Standard Deviation (SD).

## Results

A total of 60 lymph node biopsies were included in this study, of which 33(55%) belonged to males and 27(45%) to females. Of these 60 biopsies, 49(81.6%) were malignant and 11(18%) benign; distribution of cases according to diagnosis is depicted in Table 1.

**Table 1:** Distribution of Cases According to Diagnosis

Type	Diagnosis	Total No. of Biopsies	%
Benign Disorders	Reactive Follicular Hyperplasia	5	8
	Angioimmunoblastic lymphadenopathy	3	5
	Castleman’s Disease	2	3
	Sjogren’s Syndrome	1	2
Malignant Disorders	Non Hodgkin’s Lymphoma	35	58
	Hodgkin’s Disease	12	21
	Metastatic carcinoma	02	2

Age distribution of the cases showed maximum number of cases (23.3%) in the age group of 41 to 50years old, followed by 21.6% in the age group of 51 to 60years old. However, the mean age±SD was 43.5±14.9years withthe youngest patient being14years old and the oldest73years old. Out of the 60 cases, a male preponderance was noted, with 55% (33) males and 45%(27) females; the oldest and youngest patients were males.

**Figure 1:**
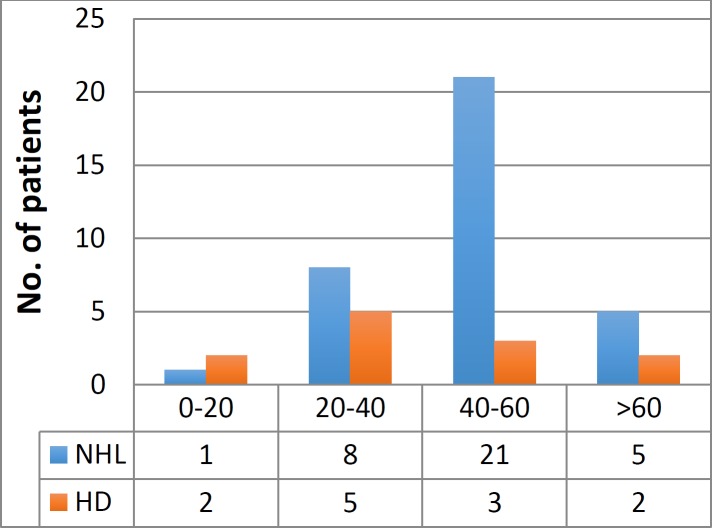
Age Distribution of Lymphoma Cases

Out of the 49 biopsies of malignant lesions, 35 (71.4%) were diagnosed as Non-Hodgkin’s Lymphoma and 12 (24.4%) as Hodgkin’s disease, with 2 (4.08%) being of metastatic carcinoma (Figure 1).

The figure shows the distribution of age in malignant lymphoma cases. Maximum number of cases (24 individuals; 40%) belonged to the age group of 40 to 60years old, including45% females and 55% males (Table 2).

The initial diagnosis for malignant lymphoproliferative disease was made using morphological criteria and a panel of monoclonal antibodies were used for subtyping these malignancies. Histopathological typing of the 35 NHL biopsies according to the Working Formulation Classification is demonstrated in Table 3.

**Table 2:** Distribution of Cases According to Age and Gender

Age Group (Years)	Males	Females	Total (%)
<20	1	3	4(6.6%)
21-30	8	4	12(20%)
31-40	4	5	9(15%)
41-50	6	8	14 (23.3%)
51-60	8	5	13(21.6%)
>60	6	2	8(13.3%)
Total	33(55%)	27(45%)	60

The intermediate group, which comprised of diffuse mixed and diffuse large cell lymphoma, was the largest group consisting 63% (22) of cases. High grade lymphomas included immunoblastic (2 of 35 biopsies) and Burkitt like lymphomas (1 of 35 biopsies). According to clinical groupings, the NHL cases were divided to indolent lymphomas (26%), moderately aggressive (3%), aggressive (68%) and highly aggressive (3%) biopsies (Table 4).

**Table 3:** Histopathological Typing of Non Hodgkin Lymphomas Biopsies According to the Working Formulation Classification

Grade	Histopathological Typing	No of Biopsies	Percentage of Biopsies
Low (n = 10)	Follicular. L	03	28%
	Small Lymphocytic L	07	
Intermediate (n = 22)	Diffuse Mixed (small & large cell) L	12	63%
	Diffuse Large Cell Lymphoma	10	
High (n = 03)	Immunoblastic L	2	9%
	Burkitt Like Lymphoma	01	

**Table 4:** Histopathological and Immuno phenotypic Subtyping of Non Hodgkin Lymphomas (Clinical Groupings)

ClinicalGroup	Histopathological Typing	No of Biopsies	Percentage of Biopsies	Immuno phenotype
Indolent	B-SLL	04	**26%**	B cell
	Maltomas	02		B cell
	Follicular cell L	02		B cell
	Mycosis Fungoides	01		T cell
Moderately Aggressive	Follicular Large Cell L	01	**3%**	B cell
Aggressive	DLBCL	13	**68%**	B cell
	ALCL	07		T cell
	PTCL	04		T cell
Highly Aggressive	Burkitt-Like Lymphoma	01	**3%**	B cell

**Abbreviations:** SLL: Small Lymphocytic Lymphoma, DLBCL: Diffuse Large B-Cell Lymphoma, ALCL: Anaplastic Large Cell Lymphoma PTCL: Peripheral T-Cell Lymphoma

**Figure 2:**
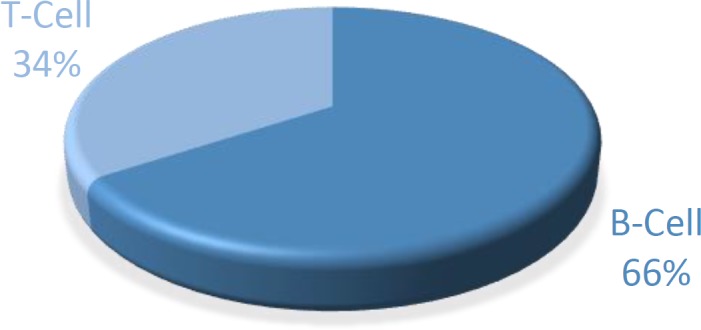
Distribution of Non Hodgkin Lymphomas cases according to Immunophenotype

Immuno phenotypic typing of the NHL_4 _biopsies showed that the majority (66%) were of B-cell type constituting (23) while 34% (12) were of T-cell type (Figure 2).

Overall, 27 (77%) were primary to the lymph nodes and 8 (23 %) were primary to the extra-nodal tissues. Immuno phenotypic subtyping of individual cases is shown in Table 4.

Genes involved in regulation of apoptosis were evaluated in NHL cases, these included p53, Bcl2andBaxalongwith proliferation antigens, such as Proliferating Cell Nuclear Antigen (PCNA).

Bax and Bcl-2 immunostains were evaluated using a 100X and 50X oil lens, in 10 fields by light microscopy. Areas containing the most uniformly stained sections were chosen for evaluation, with attempts made to avoid edge artifact. Bax and BCL-2 protein expression was quantitated by percentage of immunopositive neoplastic cells per ten fields. The amount of positive cells was graded on a scale of 1 to4; 1% to 25%, 26% to 50%, 51% to 75%, and 76% to 100% were considered grades 1, 2, 3 and 4, respectively. Only cytoplasmic immunostaining was evaluated. A BBPR was calculated for each case. The mean BBPR was found to be significantly higher in indolent lymphomas (2.64 ± 1.3) as compared to aggressive lymphomas (0.47 ± 0.9) (P<0.01). The BBPR in moderately aggressive and highly aggressive lymphomas (comprising of only one biopsy in each group) was 3 and 0, respectively.

The PCNA labeling index was calculated as a percentage of positive cells. The results are presented as mean ± SD. The Labeling index ± SD in indolent lymphomas was 26.8 ± 7.9, while in aggressive lymphomas this was 74.3 ± 11.6.

The P53 expression was absent in all biopsies of low-grade lymphoma, while weak expression was seen in high-grade lymphoma. Moderate expression was seen in 9%of biopsies of intermediate grade and 33% of high-grade lymphomas. Strong expression was seen in 18% of biopsies of intermediate grade and 33% of those of high grade lymphomas. According to International Lymphoma Study Group classification, P53 expression was absent in all cases of indolent, moderately aggressive, and highly aggressive lymphomas, moderate expression was seen in 19%of biopsies, while strong expression was seen in 28% biopsies of aggressive lymphomas. The expression of P53 and PCNA in 35 biopsies of NHLs was found to increase from low to high grade tumors, suggesting that this may be of prognostic value in these disorders.

Hodgkin’s disease was found in 12 cases out of 47 biopsies of malignant lymphoproliferative diseases. Of these 12 biopsies, on further subtyping, 2 (17%) cases were of lymphocytic predominance, 4 (33%) of nodular sclerosis and 6 (50%) of mixed cellularity (Table 5). 

**Table 5:** Histopathological Typing of Hodgkin’s disease

	Sub Typing	No of Biopsies (N = 12)	Percentage
Hodgkin’s Disease	Lymphocytic predominant	2	17
	Nodular sclerosis	4	33
	Mixed cellularity	6	50

The expression of proliferative and apoptotic related proteins were evaluated in these biopsies;Bax was frequently expressed in Hodgkin’s/Reed Sternberg cells with low expression of Bcl-2. Comparison between Bcl-2 and Bax staining showed their co-expression or absence of expression of both proteins in RS cells was statistically meaningful. To assess proliferative activity in Hodgkin’s disease, PCNA was used and indicated that the cytoplasm of Reed Sternberg (RS) cells and its variants were stained positively. Indexes of PCNA and P53 were significantly higher in patients with advanced disease than those at the early stages of the disease. Statistical analysis also led to the conclusion that these indexes could be taken into consideration as a new prognostic factor in Hodgkin’s disease**.**

## Discussion

Homeostasis in normal tissues is maintained by a balance between cell proliferation and cell death. Dysregulation of this balance, which contributes to clonal expansion of malignant cells, is a well-documented phenomenon. Both positive and negative regulators of cell growth may be involved in a neoplastic growth (8).Apoptosis is an event that leads to the death of cells without the release of harmful substances into the tissue. In mitochondria-mediated apoptotic cell death process,Bax activation irreversibly leads to cell death. The Bax promoter contains response elements for an important tumor suppressor, p53, and this affects gene expression (9).The Bcl-2 gene, a proto-oncogene that blocks apoptosis, was identified at the chromosomal breakpoint of t(14;18) bearing B-cell lymphomas. Its gene product is an anti-apoptotic molecule that modulates the mitochondrial release of cytochrome c, and the interaction of apoptosis activating factors with caspase 9 and Bax (Bcl-2 associated × protein). In adults, Bcl-2is topographically restricted to progenitor and long-lived cells. In the lymph node, Bcl-2positive cells are identified in the mantle zone of germinal centers. The Bcl-2-related protein Bax heterodimerizes in vivo with Bcl-2, and opposes its effects and acts as an accelerator of apoptosis. A preset ratio of Bcl-2/ Bax appears to determine the survival or death of cells following an apoptotic stimulus. Bax is not of prognostic significance when found alone (8).In order to clarify the role of spontaneous apoptosis of NHL, Apoptotic Indices (AI), and proliferative activity were estimated in 35 biopsies of NHL. The ratio of Bcl-2 and Bax protein expression was analyzed and correlated with the histological subtype of non-Hodgkin’s lymphomas. A significant difference in the extent of apoptosis between low and high grade NHL was noted. High-grade lymphomas had significantly more biopsies with weak expression of Bcl-2 and strong expression of Bax, with a mean score of 0.47 ± 0.9, while low-grade lymphomas had a mean score of 2.6 ± 1.3. A statistically significant difference (p<0.01) was noted in the BBPR when compared with indolent (2.6) and aggressive lymphomas (0.47). A notable trend in the BBPR among lymphoma subgroups was indicated in the present study; Bcl-2 expression was greatest in SLL and follicular lymphomas, low in high grade and absent in Burkitt’s lymphomas. These findings are consistent with the study of Siono et al.(10),who investigated the extent of apoptosis in 82 non-Hodgkin’s lymphomas and concluded that apoptosis-related genes contributed to different levels between high and low grade lymphomas. 

A high BBPR in indolent lymphomas as compared to aggressive forms has also been reported in the literature similar to the results of the current study (7).This suggests that Bax and Bcl-2 expression may be linked to the biological behavior of NHL. These findings are consistent with the research of Oltavi et al.(11), who suggested that the major influential factor in cell apoptosis is BBPR. Cells with a high BBPR tended to survive and those with low BBPR underwent apoptosis more readily. Bcl-2 expression and BBPR correlate well with biological behavior. Indolent lymphomas (SLL and FCL) have a high BBPR and conversely aggressive lymphomas have a lower BBPR.A gradual downregulation of Bcl-2 protein occurred in the current study with a transition from low to high grade NHL. Diminished expression of Bcl-2 can be explained by simultaneous upregulation of its antagonists, i.e. p53 and other pro-survival molecules rather than epigenetic mechanism such as Bcl-2 promoter hypermethylation as is seen in decreasedBcl2 expression. (12)

 Our findings revealed that Bax and Bcl-2 expression might be linked to the biological behavior of NHL. Controversial results were reported by a study (13), in which 74% of NHL had Bax immune positive tumor cells with low Bcl-2 expression. BAX expression was not of prognostic significance in univariate analysis, yet, when analyzed with Bcl-2 staining, Bax provided additional prognostic significance. The authors suggested an inverse relationship between Bcl-2 expression and cell cycle transition in a recent study with no association with staining for Bax(14). Slight variation in Bcl-2 and Bax expression among the studies might be attributable to different criteria used to define positive cases, variation in antibodies, and variable success in antigen retrieval techniques. The BCL2 protein family plays an important role in regulating the cellular program of apoptosis (15). BCL2 is overexpressed in almost all types and subtypes of hematological malignancies, indicating the importance of this molecule in disease pathogenesis and evolution(16).The over-expression of B-cl2 is a highly characteristic and specific indicator of follicular lymphoma, and B-cl2 is a potentially useful diagnostic tool in sub-classification and prognosis of low-grade B-cell lymphomas(16).Bcl-2 is the founding member of a family of apoptosis-regulating proteins whose interactions are the final regulatory step before the irreversible commitment to apoptosis. Because of this fundamental role in lymphoma pathophysiology, Bcl-2 is also an attractive target for molecular therapy (17).

Abnormal cell proliferation plays a decisive role in the development of human malignancies. The P53 is a tumor suppressor gene that maintains genomic stability either by inducing cell cycle arrest or apoptosis. It is frequently inactivated during oncogenesis and is associated with poor prognosis (18). In the current study, the expression of P53 and biopsies of NHLs was found to increase from low to high grade tumors suggesting that this may be of prognostic value in these disorders. This was in agreement with previous studies (13,19,20).The concomitant accumulation of p53 protein in high grade lymphomas may lead to mutant p53 protein binding to the transcriptional silencer within the Bcl-2 promoter and therefore downregulating Bcl-2 protein expression(21),a phenomenon that explains the pattern of Bcl2 expression in NHL. Immunohistocemical expression of P53 protein in NHL and its relationship with Proliferating Cell Nuclear Antigen (PCNA) has been reported in the literature, and the proportion of positive cases has increased from low grade non-Hodgkin’s lymphoma, with higher frequency in tumors of T cell origin. The PCNA Labeling Index (LI) was significantly lower in low grade non-Hodgkin’s lymphoma, yet, no difference was established between intermediate and high grade non-Hodgkin’s lymphoma (22). Proliferating Cell Nuclear Antigen (PCNA) is a highly conserved nuclear protein that is expressed during cell replication and DNA repair. Rabenors et al. reported that immunocytochemical detection of PCNA represents a useful tool for the study of tumor proliferation activity and has a relationship with the histological grading in NHL (23). The differences among the groups were significant; the variations inside each histological subtype was correlated with low and high grade lymphomas. Proliferating Cell Nuclear Antigen may be used as a marker of cell proliferation in clinical studies to estimate biological aggressiveness of lymphomas; its determination in intermediate grade NHL could be very useful to evaluate individual cases in this group and determine prognosis and the appropriate therapy(20,23).In the current study, the expression of P53 and PCNA in 35 biopsies of NHLs was found to increase from low to high grade tumors, suggesting that this may be of prognostic value in these disorders.

The pathogenesis of Hodgkin’s disease remains poorly understood, thus in the current study the expression of apoptotic- and proliferation-related genes were also evaluated in 12 cases of Hodgkin’s disease. Apoptotic marker Bax was frequently expressed in Hodgkin’s/Reed Sternberg (HRS) cells with low expression of Bcl-2 in the present study. It has been demonstrated that HRS cells are derived from germinal center B cells, which harbor clonally rearranged and somatically mutated immunoglobulin genes yet lack immunoglobulin expression (24).These aberrant cells usually undergo apoptosis, yet dysregulation of the Bcl-2 pathway may provide a way for the cells to escape the apoptotic program (24). Hence, the expression of bcl-2, other bcl-2 family members (e.g, bcl-XL, BAX), and apoptosis regulators are altered in HL(25).The altered expression of these proteins in HRS cells may prevent apoptosis caused by the absence of functional B-cell receptor and explain resistance to treatment-induced apoptosis and treatment failure(26).The prognostic value of Bcl-2 in CHL has been examined andBcl-2 has been found as an independent factor that predicts poor prognosis when considered together with clinical variables (27).

Frequent expression of a cell death-induced genes, Bax as compared to Bcl2, was investigated in Hodgkin’s disease by Brousset et al. (28) and compared with the immune-detection of apoptosis regulating proteins, concluding that Bax is frequently expressed in HD. This provides a potential explanation for the good chemo responses generally obtained for patient with this neoplastic disorders. Comparison between Bcl-2 and Bax staining showed a statistically significant relationship for co-expression cases or absence of expression of both proteins in RS cells in a study by Knavaros et al. (29).Reduced Bax expression was attributed to post-transcriptional regulation with lack of Bax immunosuppression, providing immunohistocemical evidence for deregulated expression of cell cycle and apoptotic related proteins, which may play a role in pathogenesis of HD**.** Indexes of PCNA and P53 were significantly higher in patients with advanced disease than in early stages of the disease in the present study. The high proliferative PCNA index and high expression of P53 and BCL-2 correlates with poor response to treatment (30).Expression of both P53 and BCL-2 proteins negatively influences overall survival and disease free survival. Statistical analysis also led to the conclusion that these indexes can be taken into consideration as a new prognostic factor in Hodgkin’s disease**. **Furthermore, p53 is essential for regulating cell death by inducing apoptosis following cell damage in response to cytotoxic agents (29).

## Conclusion

The expression of apoptotic-associated proteins (Bcl-2 and Bax) and their ratio (Bcl-2: Bax protein ratio, BBPR) was correlated with immune phenotyping and the histological grade of the tumors in the present study. The mean BBPR was significantly higher for indolent as compared to aggressive lymphomas and the mean proliferative labelling index was significantly lower in indolent as compared to aggressive lymphomas. A significant correlation was found between BBPR and the predicted biological behavior of indolent and aggressive lymphomas, indicating the important role of Bcl-2 and Bax in biological behavior of lymphomas. It is probable that the major influential factors in cell apoptosis are BBPR, and cells with high BBPR tend to survive and those with low BBPR will undergo apoptosis more readily. The P53 expression was strongly expressed in high-grade lymphomas with weak or no expression in low-grade lymphomas. The expression of P53 and PCNA was found to increase from low to high grade tumors suggesting that this may be of prognostic value in these disorders. In HD, indexes of PCNA and P53 were significantly higher in patients with advanced disease than in early stages of disease. Statistical analysis also led to the conclusion that these indexes can be taken into consideration as new prognostic factors in Hodgkin’s disease, and Bcl-2 is an independent factor that predicts poor prognosis**.**
